# Aphid performance changes with plant defense mediated by *Cucumber mosaic virus* titer

**DOI:** 10.1186/s12985-016-0524-4

**Published:** 2016-04-22

**Authors:** Xiaobin Shi, Yang Gao, Shuo Yan, Xin Tang, Xuguo Zhou, Deyong Zhang, Yong Liu

**Affiliations:** Key Laboratory of Integrated Management of the Pests and Diseases on Horticultural Crops in Hunan Province, Hunan Plant Protection Institute, Hunan Academy of Agricultural Sciences, Changsha, 410125 China; Department of Entomology, University of Kentucky, Lexington, KY 40546 USA; Longping Branch, Graduate College, Hunan University, Changsha, 410125 China

**Keywords:** *Cucumber mosaic virus*, *Myzus persicae*, Plant defense, Jasmonic acid, Salicylic acid

## Abstract

**Background:**

*Cucumber mosaic virus* (CMV) causes appreciable losses in vegetables, ornamentals and agricultural crops. The green peach aphid, *Myzus persicae* Sulzer (Aphididae) is one of the most efficient vectors for CMV. The transmission ecology of aphid-vectored CMV has been well investigated. However, the detailed description of the dynamic change in the plant-CMV-aphid interaction associated with plant defense and virus epidemics is not well known.

**Results:**

In this report, we investigated the relationship of virus titer with plant defense of salicylic acid (SA) and jasmonic acid (JA) during the different infection time and their interaction with aphids in CMV-infected tobacco plants. Our results showed that aphid performance changed with virus titer and plant defense on CMV-inoculated plants. At first, plant defense was low and aphid number increased gradually. The plant defense of SA signaling pathway was induced when virus titer was at a high level, and aphid performance was correspondingly reduced. Additionally, the winged aphids were increased.

**Conclusion:**

Our results showed that aphid performance was reduced due to the induced plant defense mediated by *Cucumber mosaic virus* titer. Additionally, some wingless aphids became to winged aphids. In this way CMV could be transmitted with the migration of winged aphids. We should take measures to prevent aphids in the early stage of their occurrence in the field to prevent virus outbreak.

## Background

Plants are constantly attacked by many kinds of plant viruses, and they have evolved extraordinarily complex mechanisms to defend themselves [1]. About 80 % plant viruses depend on insect vectors for transmission [2, 3]. At present, the triple plant-virus-vector interaction has been paid more and more attention for understanding the complex interplay of factors resulting in virus emergence. In the current study, we investigate the triple interactions between *Cucumber mosaic virus* (CMV), *Myzus persicae* Sulzer (Aphididae), and tobacco plants.

*Cucumber mosaic virus* (CMV) causes appreciable losses in vegetables, ornamentals and agricultural crops [4, 5]. CMV has a broad host range, including more than 1, 200 plant species in over 100 families [6]. CMV is transmitted by 80 species of aphids in 33 genera in a non-persistent manner [5]. The green peach aphid, *M. persicae* is one of the most efficient vectors for CMV [7], and is frequently used in transmission experiments [8].

Plants can antagonize the growth, development and preference of insect vectors directly and therefore affect virus transmission indirectly. The plant–vector interaction appears favorable to the persistent transmission, such as those described for *Barley yellow dwarf virus* (BYDV) and *Potato leaf roll virus* (PLRV), that both attract vectors to and encourage their population growth and sustained feeding on infected plants [9, 10]. Our previous results also showed that infection of *Tomato yellow leaf curl virus* (TYLCV) increased the performance of whiteflies, *Bemisia tabaci* to facilitate virus transmission [11–13]. Previous research, however, revealed the different pattern of plant-vector interaction for transmission of non-persistent viruses from that of the transmission of persistent viruses [14]. CMV-infected squash plants are poor hosts for aphid vectors, while aphids exhibited a preference for the elevated volatile emissions of infected plants. Besides, CMV infection induces changes in host palatability and quality for aphid vectors rapid dispersal following virus acquisition [15, 16]. The transmission ecology of aphid-vectored CMV has been well investigated, and interactions between viruses and aphids are key factors influencing CMV epidemics [17]. However, the detailed description of the dynamic change in the plant-CMV-aphid interaction associated with plant defense and virus epidemics was not well known.

The signaling pathways in this plant-CMV-aphid interaction influence each other through a complex network of synergistic and antagonistic interactions [18]. The phytohormones salicylic acid (SA) and jasmonic acid (JA) are known to participate in defense responses in plants [19–21]. There is considerable cross-talk between JA and SA [22–25]. In plant-insect interactions, SA induction has been confirmed to be an effective defense response against aphids and whiteflies [26, 27]. β-1, 3-glucanases is an important pathogenesis-related (PR) protein in response to pathogenic infection mediated by SA (Livne [28]; Chen [29]). Protease inhibitor is caused by JA as a result of injury (Turner [30] Zhang et al. [31]).

In the process of virus infection, whether the virus titer has a time effect on plant defense such as JA and SA in molecular and biochemical level which can regulate aphid performance is largely unknown. In this report, we investigated the relationship of virus titer with SA and JA during the different infection time and their interaction with virus vector aphids in CMV-infected tobacco plants. Our goal was to find some ecological mechanisms in the virus epidemics of CMV.

## Methods

### Plant, aphid colonies and virus culture

Tobacco plants (*Nicotiana tabacum cv. Samsum*) were grown in a potting mix (a mixture of vermiculite, peat moss, organic fertilizer and perlite in a 10:10:10:1 ratio by volume) in insect-free cages (60 × 60 × 60 cm) in a glasshouse. *Myzus persicae* (Sulzer) were raised in colonies on tobacco plants. When plants were at the 3–4 true leaf stage, they were inoculated with 5 cm^2^ of frozen stock tissue infected with CMV (stored at −80 °C). Frozen tissue was ground with 5 ml of 0.1 M potassium phosphate buffer on a cold surface. Carborundum powder was then added and the mixture was applied to surfaces of tobacco leaves using cotton swabs. Control plants were mock-inoculated in the same manner, but with healthy tobacco tissue.

### Viral load with DAS-ELISA

Virus titer was determined after 3, 6, 9, 12 and 15 days of CMV-inoculation. The identity of the virus titer was detected though DAS-ELISA using diagnostic kit (ADGEN, UK).

### JA and SA in molecular and biochemical level

The gene expression of the JA and SA signaling pathway in CMV-inoculated plants were determined at 3, 9 and 15 days post-inoculation. The JA upstream gene *OPR3* and downstream genes *COI1* and *PDF1.2* were measured. At the same time the SA upstream gene *ICS1* and downstream genes *NPR1* and *PR1* were measured. *Actin* was used as the internal reference gene [32]. Total RNA was extracted from 0.2 g of CMV-inoculated leaves, and 1.0 μg of RNA was used to synthesize the first-strand cDNA using the PrimeScript® RT reagent Kit (Takara Bio, Tokyo, Japan) with gDNA Eraser (Perfect Real Time, TaKara, Shiga, Japan). The 25.0 μl reaction system containing 10.5 μl of ddH_2_O, 1.0 μl of cDNA, 12.5 μl of SYBR® Green PCR Master Mix (TIANGEN, Beijing, China), and 0.5 μl of each primer (Table 1). Relative quantities of RNA were calculated using the comparative cycle threshold (Ct) (2-ΔΔCt) method [33]. Three biological replicates and four technical replicates were analyzed.

**Table 1:** Primer sequences used for qPCR analysis

Gene	GeneBank accession no.	Primer sequence
*OPR3*	EF467331	F: 5‘- AGGCACTATGATTTCTC-3‘ R: 5‘- GTTGATCCCATCTTTC-3‘
*COI1*	AY547493	F: 5‘- CACTTGATAATGGTGT-3‘ R: 5‘- AGGCCTTCATCGGATTCC-3‘
*PDF1.2*	X99403	F: 5‘- AACTTGTGAGTCCCAGAG-3‘ R: 5‘- GGATACCTTTCTACCACC-3‘
*ICS1*	DQ149918	F: 5‘- TTAAACTCATCATCTTCAG-3‘ R: 5‘- GGCTTCGCCGGCATTCATT-3‘
*NPR1*	AF480488	F: 5‘- GCTGTGGCATTCCTGGTT-3‘ R: 5‘- GTGAGCCTCTTGGCGATT-3‘
*PR1*	JN247448	F: 5‘- TGCCTTCATTTCTTCTTG-3‘ R: 5‘- TTAGTATGGACTTTCGCCTCT-3‘
*Actin*	AY179605	F: 5‘- AACTGATGAAGATACTCACA-3‘ R: 5‘- CAGGATACGGGGAGCTAAT-3‘

The activity of proteinase inhibitor (PI) and β-1, 3-glucanases (GUS) of CMV-inoculated leaves were determined at 3, 9 and 15 days post-inoculation. The activity of PI was determined using standard protocol [12]. The activity of GUS was determined using standard protocol [34]. GUS activity was calculated as nmoles of MU per minute per milligram of protein. Three biological replicates and three technical replicates were analyzed.

### Aphid performance

Vaseline was plastered at the culm of the tobacco to prevent aphids from escaping. After a 4 h equilibration period, 20 apterous adults of the same age, which had been starved for 4 h, were placed separately on mock-inoculated and CMV-infected tobacco plants. The mock-inoculated and CMV-infected tobacco plants with aphids were placed separately in insect-free cages (40 × 20 × 40 cm).

After 3, 6, 9, 12 and 15 days, the number of apterous and alate aphids on mock-inoculated and CMV-infected plants were count and recorded. In order not to miss any aphids, all the spaces in the cages were also checked. After each count, winged aphids were all removed by an aspirator, in order not to interfere the observation in the next time. Each experiment was repeated eight times.

To determine the longevity, aphids was collected and transferred to mock-inoculated and CMV-infected tobacco plants. Each plant was placed 20 apterous adults of the same age. The new born aphids were removed and every female was checked every day until their death, and the longevity of aphids was recorded.

### Statistical analysis

One-way ANOVA was used to compare viral titer of CMV-infected leaves and to compare relative gene expression and enzyme activity of JA and SA signaling pathway. One-way ANOVA was also used to compare number of winged aphids on CMV-infected plants. Repeated-measures ANOVAs were used to compare the number of aphids on mock-inoculated plants and CMV-infected plants. Longevity of aphids on mock-inoculated plants and CMV-infected plants were compared with *t*-tests.

## Results

### Viral load with DAS-ELISA

**Fig. 1:**
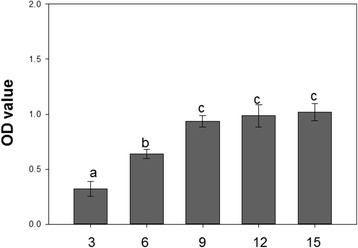
Viral load with DAS-ELISA. The virus was *Cucumber mosaic virus* (CMV). Virus titer was determined after 3, 6, 9, 12 and 15 days of CMV-inoculation. Values are means ± SE. Different letters indicate significant differences (*P* < 0.05)

Viral load differed significantly in the first 15 days (*F* = 17.462, *P* < 0.001). Virus titer grew continuously at the beginning, and then, from the 9^th^ day, virus titer remained at a relatively stable level (Fig. 1).

### JA and SA in molecular and biochemical level

**Fig. 2:**
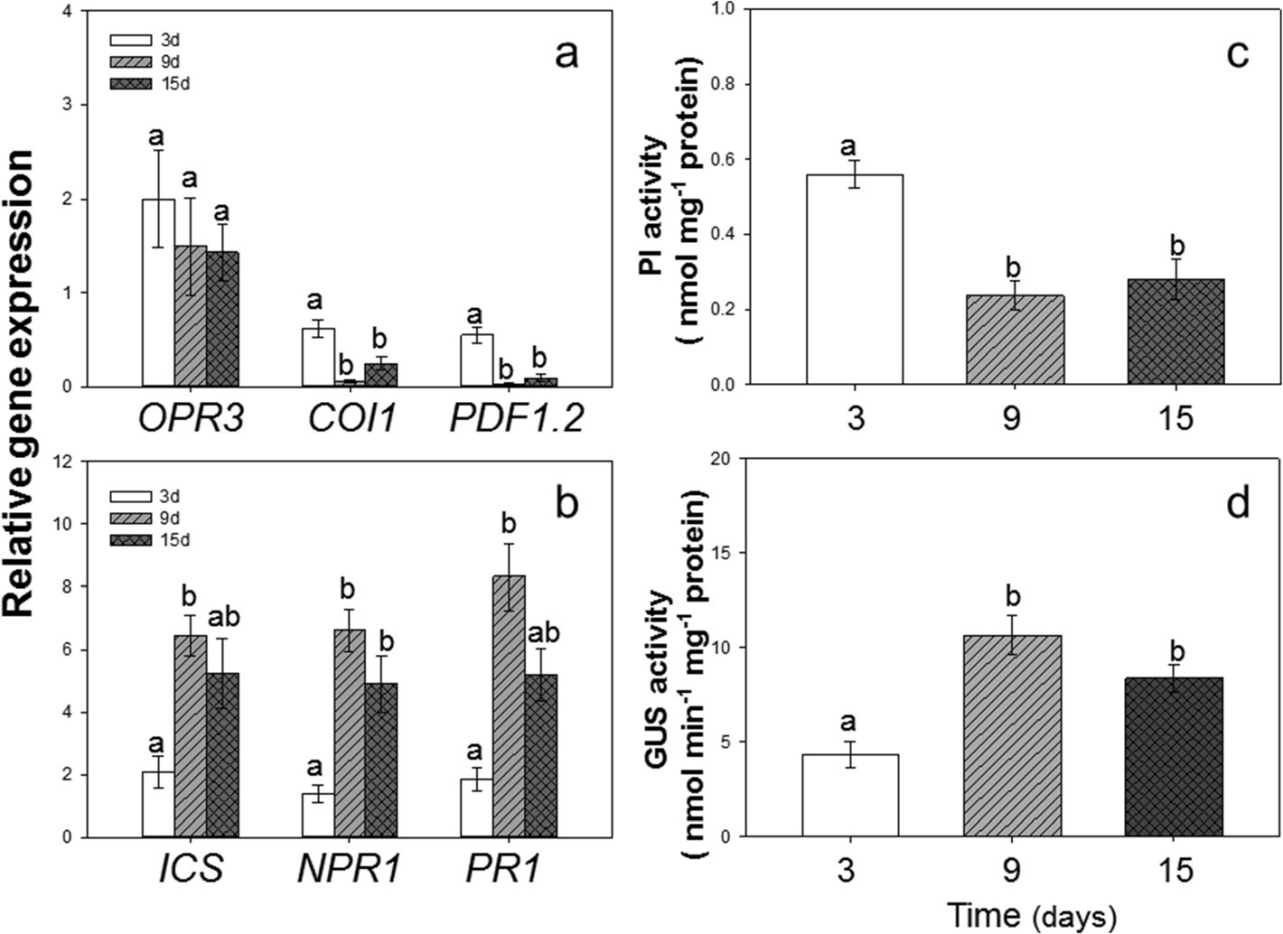
JA and SA in molecular and biochemical level. Gene expression levels and enzyme activities were determined on CMV-inoculated plants after 3, 9 and 15 days. **a** Gene expression levels of JA upstream gene *OPR3* and downstream genes *COI1* and *PDF1.2*. **b** Gene expression levels of SA upstream gene *ICS* and downstream genes *NPR1* and *PR1*. **c** Proteinase inhibitor (PI) activity. **d** β-1, 3-glucanases (GUS) activity. Values are means ± SE. Within each panel, different letters indicate significant differences (*P* < 0.05)

The expression of the JA upstream gene *OPR3* was increased, and the expression of the JA downstream genes *COI1* and *PDF1.2* was decreased. Besides, the expression of *COI1* and *PDF1.2* was number numerically lowest on the 9^th^ day (Fig. 2a). The expression of SA upstream gene *ICS1* and downstream genes *NPR1* and *PR1* was increased on CMV-inoculated plants. The SA-responsive gene expression was numerically highest on the 9^th^ day (Fig. 2b).

PI activity was reduced from 3 days to 15 days and was numerically lowest on the 9^th^ day (one-way ANOVA: *F* = 15.023, *P* = 0.005, Fig. 2c). GUS activity was increased from 3 days to 15 days and was numerically highest on the 9^th^ day (one-way ANOVA: *F* = 15.902, *P* = 0.004, Fig. 2d).

### Aphid performance

**Fig. 3:**
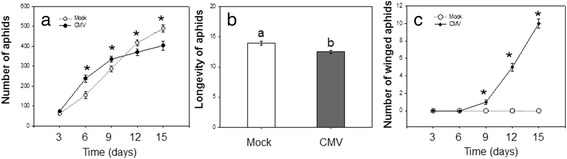
Aphid performance on CMV-inoculated and mock-inoculated plants. **a** Aphid number. **b** Aphid longevity. **c** Number of winged aphids on CMV-inoculated plants. The virus was *Cucumber mosaic virus* (CMV). CMV: CMV-inoculated plants. Mock: mock-inoculated plants. Values are means ± SE. Asterisks indicate significant differences (*P* < 0.05, **a**). Different letters indicate significant differences (*P* < 0.05, **b** and **c**)

On the third day, aphid number was similar on CMV-infected plants and mock-inoculated plants. From the 6^th^ day to the 15^th^ day, there was significant difference in aphid numbers between individuals on CMV-infected plants and mock-inoculated plants (repeated-measurement ANOVA: *F* = 2739.310, *P* < 0.001). Aphid growth rate on mock-inoculated plants only changed a little. However, aphid growth rate on CMV-infected plants slowed down gradually (Fig. 3a). The longevity of aphid on CMV-infected plants was significantly lower than on mock-inoculated plants (*F* = 0.293, *P* = 0.005; Fig. 3b). The number of winged aphids increased on CMV-infected plants from the 9^th^ day, and the number of winged aphids was highest on CMV-infected plants on the 15^th^ day (Fig. 3c).

## Discussion

Our results demonstrate that there is a clear link between the aphid number, virus titer and plant defense. The number of aphids on virus-infected plants significantly increased in the beginning, but the number declined significantly after the virus titer maintained a certain level. Besides, the longevity of aphids on CMV-infected plants was lower than that on mock-inoculated plants. Our results show that in the early infection of CMV, infected plants can promote the growth and development of aphids. However, when the viral titer remained stable in plants, the growth of aphid was decreased. One previous result showed that survival of *M. persicae* was lower on CMV-infected tobacco, as compared to mock-inoculated plants within 14 days [35]. Another previous result showed that performance of *M. persicae* was dramatically reduced on CMV-infected plants within 15 days [14]. Combined with our results, we can find that aphid performance on CMV-infected plants is time-dependent.

Our results also showed that plant defense changed with the increase of virus titer. In the initial stages of CMV infection, only the expression of JA upstream gene *OPR3* was induced to a higher level. However, the SA-relative genes were induced a little. On the 9^th^ day when the viral titer was highest, the expression of SA-responsive genes such as *NPR1* was highest, and the expression of JA downstream genes was lowest. *NPR1* has been reported to play a key role in the regulation of SA and JA antagonism [36]. For example, the infection of necrotrophic fungus *Botrytis cinerea* activates SA signaling via a tomato *NPR1* homolog to exploit the antagonistic crosstalk between SA and JA signaling [37]. The 2b protein of CMV targets *NPR1* to exploit SA-JA antagonism [38, 39]. Our previous also showed that infection of TYLCV induced the *NPR1* expression and reduced the JA downstream gene expression [12, 27]. Here we found that the *NPR1* and *PR1* both were induced by CMV infection and they play important roles in the antagonistic crosstalk between the SA and JA pathways. Therefore, CMV infection induced SA-regulated gene expression and disrupted JA-regulated gene expression, which is consistent with previous results [15, 35].

Proteinase inhibitor plays an important role in resisting insect herbivores and has been reported to be related with JA [31]. β-1, 3-glucanases is also an important enzyme that is involved in response to salicylic acid (SA) [40]. In our results PI activity was reduced while GUS activity was increased, which is consistent with the expression change of JA and SA relative genes.

According to our results, compared with the mock-inoculated plants, plant defense on CMV-infected plants in its early stage was low, and the aphid number increased rapidly. However, when the viral titer remained stable in plants, plant defense especially SA-responsive genes were induced to a higher level. SA can have neutral or negative effects on the growth of aphids [41]. Avila et al. [42] showed that *FAD7* enhances plant defenses against aphids that are mediated through SA and *NPR1*. SA induction has been confirmed to be an effective chemical defense response against aphids [26]. In our research SA were induced by the increase of virus titer and therefore the growth of aphid was decreased.

Another possibility to consider is that plant quality is changed by infection of CMV. Previous research showed that CMV infection reduces the host palatability and quality, and the phloem sap quality is also reduced [15]. Combined with our results, it can be found that with the increase of viral titer, aphid performance can be reduced due to the reduction of plant physiology and morphology.

We also found that number of winged aphids increased with the increase of viral titer. Many factors, such as environmental conditions, aphid density and host plant quality, may influence wing production [43]. For example, a decrease in plant quality can trigger wing induction in some aphid species [44]. Here, we show that winged *M. persicae* on virus-infected leaves are more than on mock-inoculated leaves after 9 days, although the number of aphids on virus-infected leaves is lower than on mock-inoculated leaves. Therefore, we consider it unlikely that our result is caused by aphid density. The possible explanation is that plant defense is induced by CMV infection therefore plant quality is changed, which is consistent with previous results that plant quality decreases under infection of non-persistent viruses to promote aphid migration [45]. In our results we found that plant quality decreases under infection of non-persistent viruses and then the wingless aphids become to winged aphids.

## Conclusions

We find some ecological mechanisms in the virus epidemics of CMV. The aphid performance changed with virus titer and plant defense on CMV-inoculated plants. At first, plant defense was low and aphid number increased gradually. The plant defense of SA signaling pathway was induced when virus titer was at a high level, and aphid performance was correspondingly reduced. Additionally, the wingless aphids became to winged aphids. CMV could be transmitted with the migration of winged aphids. We should take measures to prevent aphids in the early stage of their occurrence in the field to prevent virus outbreak. The physiological, biochemical and molecular mechanisms of wing production need to be further investigated.
